# Editorial for the Special Issue “Advances in the Development of Anticancer Drugs”

**DOI:** 10.3390/ijms25010641

**Published:** 2024-01-04

**Authors:** Jean Fotie

**Affiliations:** Department of Chemistry and Physics, Southeastern Louisiana University, Hammond, LA 70402-0878, USA; jean.fotie@southeastern.edu; Tel.: +1-985-549-5112

As mortality rates for other leading causes of death, such as stroke and coronary heart disease, decline in many parts of the world, cancer is becoming the leading cause of death worldwide, with the number of yearly new cases expected to rise to about 30 million by 2040 [1,2]. Despite advancements in prevention, diagnosis, and treatment, conventional approaches such as radiotherapy and chemotherapy still face limitations due to their lack of selectivity, leading to high toxicity and resistance [3]. However, significant breakthroughs towards the development of more selective and targeted treatments, including tumor-targeting chemotherapies, antibody–drug conjugates (ADCs), peptide–drug conjugates (PDCs), stem cell therapy, and immunotherapy, are expected to improve the situation, although these new approaches are still suffering from a high number of treatment failures [4,5]. Furthermore, the emergence of genomic techniques, specifically whole-exome sequencing (WES), could be a game-changer, enabling the identification of actionable gene mutations and novel cellular targets, and leveraging tumor-associated antigens to selectively deliver active agents to specific targets [6]. Additionally, DNA editing methods like CRISPR technology are expected to significantly advance treatments for various diseases, including cancer [7]. More importantly, recent breakthroughs in artificial intelligence (A.I.) and machine learning hold great promise in revolutionizing cancer therapy in the near future, as they are sure to enable the development of safer and more effective treatment options. Despite these innovative approaches, the enduring significance of small molecules in cancer prognosis, management, and treatment should not be overlooked. In fact, the recent trends in medicinal chemistry could surely rejuvenate this traditional treatment approach, as it is well known and proven efficient in introducing new chemotherapeutic molecules into the anticancer drug pipeline or repurposing clinically approved molecules from other diseases. As such, this Special Issue includes original articles and reviews, highlighting the evolving landscape of cancer treatment while providing insights into innovative approaches in cancer therapies through structure–activity relationship investigations; in silico, in vitro, and in vivo studies; and the exploration of potential therapeutic targets and molecular mechanisms. The current editorial highlights key findings and revelations derived from the published manuscripts.

As light-activated ruthenium complexes have demonstrated significant promise in targeting cancer cells, either through singlet oxygen generation in photodynamic therapy (PDT) or by producing toxic species via photoactivated chemotherapy (PACT) [8,9], Papish and collaborators [10], in their research article, investigate the influence of the position of hydroxy groups and π-expansion on the luminescence and photocytotoxicity of ruthenium complexes with protic ligands. The primary focus of their study is photodynamic therapy, emphasizing the correlation between singlet oxygen production and other photophysical properties, including photoluminescence quantum yield and photoluminescence lifetime. Among the protic ruthenium complexes studied, three exhibited significant phototoxicity indices. The study underscores a robust correlation between high singlet oxygen quantum yields for these complexes and a prolonged lifetime for the excited state. Overall, the collected data provide important insights into the photocytotoxicity of the most active complexes, suggesting the potential involvement of multiple protonation states during cell penetration and the generation of singlet oxygen species.

Morak-Młodawska and colleagues [11], in their research communication, investigate the pharmacodynamic and pharmacokinetic properties of 1,9-diazaphenothiazines, a family of compounds known to display promising in vitro anticancer potential. The study explores parameters such as lipophilicity, adsorption, distribution, metabolism, excretion (ADME), and toxicological properties that influence the bioavailability of these compounds. The ADME properties are explored using the SwissADME server, and the molecular targets are investigated using the SwissTargetPrediction server. In the process, various filters, including Lipinski’s, Ghose’s, and Veber’s, are employed to assess the bioavailability of different derivatives and analogs of 1,9-diazaphenothiazines.

In order to address the pivotal issues of resistance to traditional chemotherapy and the chemoresistant metastatic relapse’s contribution to treatment failure and poor cancer prognosis, understanding the mechanisms by which cancer cells navigate chemotherapy-induced cell death and develop resistance is not only critical for enhancing patient survival rates but also imperative for advancing more effective therapies. In their concise review, Perán and colleagues [12] delineate the methodology and protocol employed to induce drug chemoresistance in cancer cell lines. They subsequently delve into the exploration of the defense mechanisms activated by tumor cells to elude common chemotherapy agents. Their primary focus centers on treatments for high-incidence tumors, particularly DNA-damaging drugs such as gemcitabine, 5-fluorouracil, cisplatin, and doxorubicin. The authors highlight that cancer cell lines employ various strategies, including the alteration of drug influx/efflux, enhanced drug metabolic neutralization, improved DNA repair mechanisms, and the inhibition of apoptosis-related cell death, in the development of chemoresistance. Throughout the discussion, Toledo et al. [12]. examine the roles of p53 and reactive oxygen species levels, with a specific focus on cancer stem cells and the residual cell population post-chemotherapy. The exploration encompasses diverse mechanisms contributing to increasing drug resistance, such as epithelial–mesenchymal transition, fortified DNA repair machinery, and the capability of cancer cells to evade apoptosis mediated by BCL2-family proteins.

Pereira and colleagues [13] employ cheminformatics and bioinformatics tools in their review to analyze extensive datasets encompassing molecules, gene expression, and protein–protein interactions. Their objective is to identify potential biomarkers that can serve in predicting the response to PD-1/PD-L1 inhibitors, underscoring the necessity for computational approaches in the realm of immune oncology therapies. Taking a data-driven approach, the authors aim to pinpoint potential targets for PD-1/PD-L1 immune checkpoint inhibitors (ICIs) and their involvement in the innovative development of drug candidates, including antibodies, peptides, and small molecules. Their exploration delves into various facets of computer-aided drug design approaches, encompassing ligand-based virtual screening processes, molecular docking, homology modeling, and molecular dynamics simulation methodologies. While maintaining a primary focus on the search for PD-1/PD-L1 ICIs, Sobral et al. [13]. compile a comprehensive list of recent databases and web tools utilized in the context of cancer and immunotherapy. Throughout this process, they investigate the role of molecular modeling and simulation techniques in predicting the effects of point mutations on the Ab–antigen interaction. Leveraging small molecules, proteins, and antibodies, the authors examine the binding mode and binding affinity of the PD-1/PD-L1 immune checkpoint.

Concluding this Special Issue, our research group [14], supported by around 268 cited references and over 336 carefully selected illustrative chemical structures, thoroughly explores the pivotal role of oxime and oxime ether moieties in augmenting the physicochemical and anticancer properties of diverse molecular frameworks. The analysis encompasses the significant contributions of these functional groups in the development of strategies for anticancer molecular design, modulation of biological activities, computational modeling, and structure–activity relationship studies. The review delves into the involvement of molecular dynamics and docking studies in elucidating the mechanism of action and determining the potential drug targets of compounds featuring oxime and oxime ether moieties. A key takeaway from this comprehensive examination is that, in many cases, oximes and oxime ethers are entirely responsible for the observed properties, while in others, they enhance the inherent anticancer properties of the scaffold to which the functional group is attached. Moreover, the addition of these functional groups appears to exert a substantial influence on the pharmacokinetic and pharmacodynamic profiles, as well as the drug-likeness of the resulting molecules. In essence, the review consolidates a diverse array of scaffolds bearing oxime or oxime ether functional groups, showcasing their inhibitory, interacting, or interfering capabilities with key markers in cancer pathogenesis and malignancy. 

Overall, this Special Issue showcases a well-balanced collection of relevant topics in the thriving and competitive field of anticancer drug design and development, and can serve as a valuable resource to oncologists, biologists, and medicinal chemists within the readership of this journal.

## References

[1] Li C., Lei S., Ding L., Xu Y., Wu X., Wang H., Zhang Z., Gao T., Zhang Y., Li L. (2023). Global burden and trends of lung cancer incidence and mortality. Chin. Med. J..

[2] Morgan E., Arnold M., Gini A., Lorenzoni V., Cabasag C.J., Laversanne M., Vignat J., Ferlay J., Murphy N., Bray F. (2023). Global burden of colorectal cancer in 2020 and 2040: Incidence and mortality estimates from GLOBOCAN. Gut.

[3] Tonyali S., Haberal H.B., Sogutdelen E. (2017). Toxicity, adverse events, and quality of life associated with the treatment of metastatic castration-resistant prostate cancer. Curr. Urol..

[4] Patwekar M., Sehar N., Patwekar F., Medikeri A., Ali S., Aldossri R.M., Rehman M.U. (2024). Novel immune checkpoint targets: A promising therapy for cancer treatments. Int. Immunopharmacol..

[5] Xing L., Lv L., Ren J., Yu H., Zhao X., Kong X., Xiang H., Tao X., Dong D. (2023). Advances in targeted therapy for pancreatic cancer. Biomed. Pharmacother..

[6] Mendelaar P.A.J., Smid M., van Riet J., Angus L., Labots M., Steeghs N., Hendriks M.P., Cirkel G.A., van Rooijen J.M., Tije A.J.T. (2021). Whole genome sequencing of metastatic colorectal cancer reveals prior treatment effects and specific metastasis features. Nat. Commun..

[7] Misra G., Qaisar S., Singh P. (2024). CRISPR based therapeutic targeting of signaling pathways in breast cancer. Biochim. Biophys. Acta Mol. Basis Dis..

[8] Monro S., Colón K.L., Yin H., Roque J., Konda P., Gujar S., Thummel R.P., Lilge L., Cameron C.G., McFarland S.A. (2019). Transition metal complexes and photodynamic therapy from a tumor-centered approach: Challenges, opportunities, and highlights from the development of TLD1433. Chem. Rev..

[9] McFarland S.A., Mandel A., Dumoulin-White R., Gasser G. (2020). Metal-based photosensitizers for photodynamic therapy: The future of multimodal oncology?. Curr. Opin. Chem. Biol..

[10] Oladipupo O.E., Prescott M.C., Blevins E.R., Gray J.L., Cameron C.G., Qu F., Ward N.A., Pierce A.L., Collinson E.R., Hall J.F. (2023). Ruthenium complexes with protic ligands: Influence of the position of oh groups and π expansion on luminescence and photocytotoxicity. Int. J. Mol. Sci..

[11] Morak-Młodawska B., Jeleń M., Martula E., Korlacki R. (2023). Study of Lipophilicity and ADME Properties of 1,9-Diazaphenothiazines with Anticancer Action. Int. J. Mol. Sci..

[12] Toledo B., González-Titos A., Hernández-Camarero P., Perán M. (2023). A Brief review on chemoresistance; targeting cancer stem cells as an alternative approach. Int. J. Mol. Sci..

[13] Sobral P.S., Luz V.C.C., Almeida J.M.G.C.F., Videira P.A., Pereira F. (2023). Computational approaches drive developments in immune-oncology therapies for pd-1/pd-l1 immune checkpoint inhibitors. Int. J. Mol. Sci..

[14] Fotie J., Matherne C.M., Mather J.B., Wroblewski J.E., Johnson K., Boudreaux L.G., Perez A.A. (2023). The fundamental role of oxime and oxime ether moieties in improving the physicochemical and anticancer properties of structurally diverse scaffolds. Int. J. Mol. Sci..

